# Interaction of Lead and Cadmium Reduced Cadmium Toxicity in *Ficus parvifolia* Seedlings

**DOI:** 10.3390/toxics11030271

**Published:** 2023-03-16

**Authors:** Yan Li, Xiaomao Cheng, Chengcheng Feng, Xiaoxia Huang

**Affiliations:** Southwest Landscape Architecture Engineering Research Center of National Forestry and Grassland Administration, College of Landscape Architecture and Horticulture Sciences, Southwest Forestry University, Kunming 650224, China; yanli@swfu.edu.cn (Y.L.);

**Keywords:** *Ficus parvifolia* seedlings, lead and cadmium, antagonism effect, Cd uptake and accumulation

## Abstract

Potentially toxic elements (PTEs) pollution occurs widely in soils due to various anthropogenic activities. Lead (Pb) and cadmium (Cd) coexist in soil frequently, threatening plant growth. To explore the interaction effect between Pb and Cd in *Ficus parvifolia* and the response of plant physiological characteristics to Pb and Cd stress, we designed a soil culture experiment. The experiment demonstrated that Pb stress improved leaf photosynthesis ability, while Cd stress inhibited it. Furthermore, Pb or Cd stress increased malonaldehyde (MDA) content, but plants were able to reduce it by increasing antioxidant enzyme activities. The presence of Pb could alleviate Cd phytotoxicity in plants by inhibiting Cd uptake and accumulation as well as increasing leaf photosynthesis and antioxidant ability. Pearson correlation analysis illustrated that the variability of Cd uptake and accumulation between Pb and Cd stress was related to plant biomass and antioxidant enzyme activities. This research will offer a new perspective on alleviating Cd phytotoxicity in plants.

## 1. Introduction

Potentially toxic elements (PTEs) pollution has become one of the most severe environmental problems around the world [1]. Lead (Pb) and cadmium (Cd) are common elements in PTEs pollution, which result from mining, traffic emission, and overusing pesticides and fertilizers [2,3]. Pb and Cd are highly toxicity and non-degradable [4,5], impacting plant growth and threatening human health [6,7]. A large number of studies have demonstrated that Pb and Cd pollution hurts plant growth, photosynthesis, membrane structure, and their antioxidant system [8,9,10]. The growth and physiology response of plants to Pb and Cd stress is different due to plant species, metal concentrations, and tolerance [11]. Interestingly, Pb and Cd do not exist independently within the soil but coexist [12]. Previous studies have confirmed that the interaction effect between Pb and Cd is synergism and antagonism [13]. Thus, the interaction effect between the two PTEs has either a positive or negative effect on plants. Scholars have discovered that the addition of Zn alleviated Cd toxicity to tall fescue (*Festuca arundinacea*) [14]. Murtaza et al. [15] found that the combined negative impacts of Pb and Cd stress on physiological parameters was significantly higher than that of Pb or Cd in individuals. Nevertheless, the mechanism of Pb and Cd interaction is intricate [13]. At the same time, only a few studies focus on the photosynthesis and antioxidant enzyme response of plants to combined Pb and Cd stress. Therefore, it is necessary to further analyze and compare the change of physiological characteristics under individual and combined Pb and Cd stress in *Ficus parvifolia* seedlings.

In the current literature, the remediation of PTEs-contaminated soil has received widespread attention. Many scholars have put forward methods involving physical, chemical, biological, and combinatorial remediation and phytoremediation technology [16]. Phytoremediation uses plants to repair PTEs contamination in soil [17]. This approach attracts extensive attention due to its low cost, high efficiency, and sustainability [17]. According to the accumulation and translocation characteristics of PTEs in plants, phytoremediation could be divided into phytoextraction, phytostabilization, and so on [18]. Phytostabilization takes advantage of the restraint of PTEs mobility and bioavailable in soils through plant roots to reduce the effect of PTEs on soil [19]. Phytoextraction takes advantage of PTEs absorbed by the roots and transfers them to harvestable leaves to reduce the content of PTEs in the soil [20]. Therefore, the ability to absorb and transfer PTEs in plants plays an important role in phytoremediation technology. Moreover, the substrate of pH values, organic matter content, cation exchange capacity (CEC), and other soil properties are important factors affecting metal bioavailability and mobility [18]. Previous studies [21,22] revealed that the organic compounds could combine with functional groups and Cd to form stable complexes, thereby reducing the bioavailability of Cd. Plants with fast growth, high biomass production, and deep root systems can remediate contaminated soil more effectively [11,23]. For example, the object of this study, *F. parvifolia*, has large biomass production and a developed root system. Furthermore, *F. parvifolia* has a strong tolerance to PTEs among landscaping plants in southwest China [24]. Currently, the literature about *F. parvifolia* is focused on plant diseases, insect pests, and medicines [25,26]. The effects of Pb and Cd pollution on physiological characteristics and the ability of this plant to remediate PTEs contaminated soil are rarely reported. Hence, it is essential to determine the accumulation and translocation capacity of *F. parvifolia* under Pb and Cd stress. It could provide the basis for plant selection in the remediation of PTEs-contaminated soil. 

A large number of studies report the interaction effect between Pb and Cd on plants in terms of antagonism and synergism. However, how the presence of PTEs alleviates or aggravates the toxicity of metals to plants is rarely investigated. Therefore, the present study aims to unravel the interaction effect between Pb and Cd in *F. parvifolia* seedlings as well as the response of plant physiological characteristics to Pb and Cd stress. Based on previous studies, we speculated that (1) the negative influence of Pb and Cd in combination would be higher than that in the individual, and (2) the presence of Pb may inhibit Cd uptake by plants, while the presence of Cd may promote Pb uptake by plants.

## 2. Materials and Methods

### 2.1. Experimental Materials

*F*. *parvifolia* seedlings were selected as experiment materials and procured from the nursery garden of Southwest Forestry University, Kunming, China. One-year *F*. *parvifolia* seedlings with uniform growth and no pests or diseases were transplanted into 5 L plastic pots containing a 5.5 kg soil mixture. The soil mixture was composed of red loam, perlite, and peat with a volume ratio of 3: 3: 2. Red loam (0–25 cm) was collected from the nursery garden, Southwest Forestry University; perlite and peat were procured from Kunming Yuanbo Flower and Bird Market. Before transplanting, the red loam was air-dried by the natural wind in the laboratory and then adequately mixed with perlite and peat. At the same time, we measured the physicochemical property of the soil mixture, the result of which is shown below (Table 1). The plastic pots with plants were set in a greenhouse with an ambient temperature between 20–30 °C (day) and 9–18 °C (night) and relative humidity between 35% and 80% in a natural photoperiod.

### 2.2. Experimental Design

The experiment began on 6 June and ended on 6 September, for which we adopted a two-factor completely random design. Experiments were designed based on the standard of soil pollution risk control for construction land in China (GB36600-2018) to obtain nine treatment groups (Table 2). The standard states that soil of development land with Pb and Cd levels above 400 and 20 mg·kg^−1^, respectively, needs to be assessed for human health risks. The Pb and Cd were added to the plastic pots in the form of lead nitrate (Pb (NO_3_)_2_) solution and cadmium chloride (CdCl_2_) solution, respectively. We treated the seedlings with different concentrations of Pb and Cd solution and after the one-year seedlings were transplanted into plastic pots. Then, 20 mL of Pb solution or Cd solution was poured onto the soil of treatment groups one time every two days. Similarly, 20 mL of the mixed Pb and Cd solution was poured onto the soil one time every two days. An equal volume of deionized water was added to the soil of the control treatment group. During the treatment, the pre-prepared solution was poured equally over the soil surface, and the solution lost from the soil was collected by a tray at the bottom and poured back onto the soil immediately. Each treatment consisted of 10 plants in their pots.

### 2.3. Determination Methods

#### 2.3.1. Biomass

The harvested plants were separated into root, stem, and leaf segments and carefully washed with distilled water to remove the Pb and Cd ions adsorbed to their surface. The plant segments were then dried using filter paper, oven-dried at 105 °C for 15 min, and dried at 70 °C for 48 h to obtain the dry weight (DW).

#### 2.3.2. Photosynthetic Characteristics and Chlorophyll Fluorescence Parameters

Mature leaves in the same position and fully expanded upper portions of the plant were selected to determine photosynthetic characteristics. The net photosynthetic rate (*P*_n_), stomatal conductance (*G*_s_), transpiration rate (*T*_r_), and intracellular CO_2_ concentration (*C*_i_) were tested using an LI-6400 portable photosynthesis apparatus (Li-Cor Inc, Nebraska, USA) between 8:00 and 11:00 a.m. Photosynthetic active radiation (PAR) was controlled at 1400 μmol·m^−2^·s^−1^; the temperature and relative humidity were 25 °C and 55–75%, respectively. Each treatment was repeated 5 times.

An IMAGING-PAM M-series fluorometer (WALZ) measured chlorophyll fluorescence [27]. The leaves of the tested plants were placed in the dark for 30 min, then the initial fluorescence (*F*_0_) was measured after exposure to 0.5 μmol·m^−2^·s^−1^ light. Maximum fluorescence (*F*_m_) was induced by a 2700 μmol·m^−2^·s^−1^ saturated light pulse (pulse time: 0.8 s), and the light intensity was 186 μmol·m^−2^·s^−1^. The leaves to be tested were placed on a sample table, and multiple AOI with diameters of 1 cm were selected. The kinetics curves of chlorophyll fluorescence parameters were displayed in the “Kinetics” window of the software. Maximum photochemical quantum yield (*F*_v_/*F*_m_), actual photochemical quantum yield (Y(II)), non-photochemical quenching coefficient (NPQ), photochemical quenching coefficient (*q*_P_), and electron transport rate (ETR) were directly derived from the apparatus.

#### 2.3.3. Malondialdehyde (MDA) Content and Antioxidant Enzyme Activities 

The detection of MDA content was based on the method of Hodges et al. [28]. First, 0.2 g leaves were weighed, and 5 mL of trichloroacetic acid (TCA) was added to the leaves. Thereafter, grinding was carried out, and the samples were centrifuged at 12,000 r·min^−1^ for 10 min. Subsequently, 2 mL of supernatant was extracted, and 2 mL of thiobarbituric acid (TBA) solution was added. After reaction completion, the test tube containing the reaction solution was placed in boiling water for 30 min and cooled in an ice bath rapidly before being centrifuged at 10,000 r·min^−1^ for 10 min. Finally, the absorbance values of the supernatant at 600, 532, and 450 nm were determined by spectrophotometer (TU–1901, Beijing Puchan Universal Instrument Co. LTD, Beijing, China).

The determination of superoxide dismutase (SOD) was based on the method of Giannopolitis and Ries [29]. The reaction solution consisted of 50 mmol·L^−1^ phosphoric acid buffer (pH7.8) containing 13 mmol·L^−1^ methionine, 75 μmol·L^−1^ nitro tetrazolium blue chloride, 16.7 μmol·L^−1^ riboflavin, and 0.1 mmol·L^−1^ Ethylene Diamine Tetraacetic Acid (EDTA). The absorbance value was measured at 560 nm. A unit of SOD activity was 1 mg protein in reaction solution with a 50% inhibition rate. 

The peroxidase (POD) activity was determined by referring to the method of Lin and Wang [30]. First, 80 μL of enzyme extract solution was added to 3 mL of enzyme reaction solution (50 mmol·L^−1^ Na_2_HPO_4_-NaH_2_PO_4_ buffer solution including 3 mmol·L^−1^ guaiacol and 2.5 mmol·L^−1^ H_2_O_2_) and mixed thoroughly. The absorbance value at 470 nm was read on a UV–visible spectrophotometer every 2 s within 2 min. The POD activity was calculated using the molar extinction coefficient (39.4 mmol·L^−1^·cm^−1^).

The ascorbate peroxidase (APX) activity was determined using the method of Knörzer et al. [31]. The absorbance value at 290 nm was read on the UV–visible spectrophotometer every 30 s for 3 min.

The catalase (CAT) activity was determined using the method of Aebi [32]. The absorbance value was read on the UV–visible spectrophotometer at 290 nm.

#### 2.3.4. Pb and Cd Accumulation and Translocation Characteristics in Plant 

Each plant segment was weighed before being ground and sifted (1 mm). Thereafter, 3 g of the sample was placed in a 25 mL porcelain crucible and carbonized over low fire. The ash was then transferred to a muffle furnace set to 500 °C for 4 h and was cooled down and removed. The resultant ash was then transferred to a 50 mL tube with a constant volume of 3% nitric acid. The blank solutions were prepared at the same time. The processed solution was analyzed by atomic absorption spectroscopy, and the samples with high concentrations were diluted. The Cd content in roots and leaves was calculated using standard curves [33]. The calculation method of bio-concentration factor (BCF) and translocation factor (TF) of Pb and Cd were based upon the method of Yadav et al. [34] and the equation as follows:

BCF of Pb and Cd = [Pb or Cd content (root, stem, leaf)/Pb or Cd content in soil]

Note: Pb or Cd content in soil = [the amount of Pb, Cd solution added (mg)/the weight of soil per pot (kg)]

TF of Pb and Cd = [Pb or Cd content in the shoot (stem + leaf)/Pb or Cd content in root].

#### 2.3.5. Physicochemical Characteristics of the Experimental Soil

Five soil samples from the nursery garden were placed in a box and brought back to the laboratory for analysis. Then, soil samples were air-dried for 7 days and sieved. The soil total nitrogen was measured by using the standard Kjeldahl digestion method [35]. Available soil phosphorous was measured by the molybdenum antimony colorimetric method [35]. Soil potassium was measured by the method of Lu et al. [36]. The soil pH was measured by potentiometry method with a 1: 2.5 soil: water ratio (*w/v*) [37]. The soil organic matter and organic carbon were measured by dichromate volumetric digestion and external heating method [37]. The content of Pb and Cd was measured by the method of Zhou et al. [33].

### 2.4. Statistical Analysis

SPSS 19.0, Microsoft Excel 2010, and Origin 2022b were used to analyze data and plot charts. All data were displayed as the mean ± standard error (SEs) of five replicates. The differences among different treatments were compared by two-way ANOVA. Multiple comparisons between the means were performed by Duncan’s test. *p* < 0.05 was considered a significant difference.

## 3. Results

### 3.1. Biomass of F. parvifolia Seedlings under Pb and Cd Stress

As shown in Table 3, there were no significant impacts on biomass in *F. parvifolia* under Pb stress. However, the leaf biomass significantly differed between Pb2 and Pb4 treatments. Conversely, it was found that Cd stress significantly impacted plant biomass. For example, compared with CK, root biomass was reduced by 39.4% and 44.7% under Cd stress, respectively. In addition, biomass under combined Pb and Cd treatments was higher than that under individual Cd treatments. The plant biomass under the Pb2Cd4-treatment group was significantly higher than that under the Cd4-treatment group. Moreover, the biomass in plant root, leaf, and total under combined treatments was decreased when compared to individual Pb treatments. In addition, the interaction between Pb and Cd had no significant impact on biomass (*p* > 0.05). 

### 3.2. Photosynthetic Parameters of F. parvifolia Seedlings under Pb and Cd Stress 

From Table 4, the response of *F*. *parvifolia* photosynthetic characteristics to Pb and Cd stress is clear. Compared with CK, the values of *P*_n_, *G*_s_, and *T*_r_ had a marked increase under the Pb4 treatment, while they showed a marked decrease under the high-Cd-concentration treatment. However, the *C*_i_ value changed little under Pb or Cd stress. Pb and Cd combination treatments were capable of alleviating photosynthetic injury caused by individual Cd stress. The *P*_n_ value under the Pb4Cd4-treatment group was significantly higher than that under the Cd4-treatment group. However, Pb and Cd in combination may intensify Pb toxicity when compared to individual Pb treatments. *P*_n_, *G*_s_, and *T*_r_ values under Pb2Cd2- and Pb2Cd4-treatment groups were lower than that of the Pb2 treatment. The maximum value of *P*_n_, *G*_s_, and *T*_r_ among all treatment groups was recorded in the Pb4-treatment group, while the minimum value was recorded in the Cd4-treatment group.

As shown in Table 5, it was found that *F*_v_/*F*_m_, Y(II), and ETR values significantly decreased under the Pb2, Cd2, and Cd4 treatments when compared to CK. However, the NPQ value significantly increased under the high-Cd-concentration treatment when compared to CK treatment. Furthermore, *q*_p_ and ETR values under combined Pb and Cd stress were higher than those under individual Cd stress and lower than those under individual Pb stress. The measured *q*_p_ and ETR values under the Pb2Cd4 and Pb4Cd4 treatments were higher than those under the Cd4 treatment, while *q*_p_ and ETR values under the Pb2Cd2 and Pb2Cd4 treatments were remarkably lower than those under the Pb2 treatment. Aside from NPQ, the maximum values for chlorophyll fluorescence parameters across Pb and Cd combined treatments were observed under the Pb4Cd2 treatment, while the minimum values of *F*_v_/*F*_m_, Y(II), *q*_p_, and ETR appeared under the Pb2Cd4- or Pb4Cd4-treatment groups. Moreover, the interaction between Pb and Cd had a significant impact on chlorophyll fluorescence parameters apart from *q*_p_. 

### 3.3. MDA Content and Antioxidant Enzyme Activities Change of F. parvifolia Seedlings under Pb and Cd Stress

**Figure 1:**
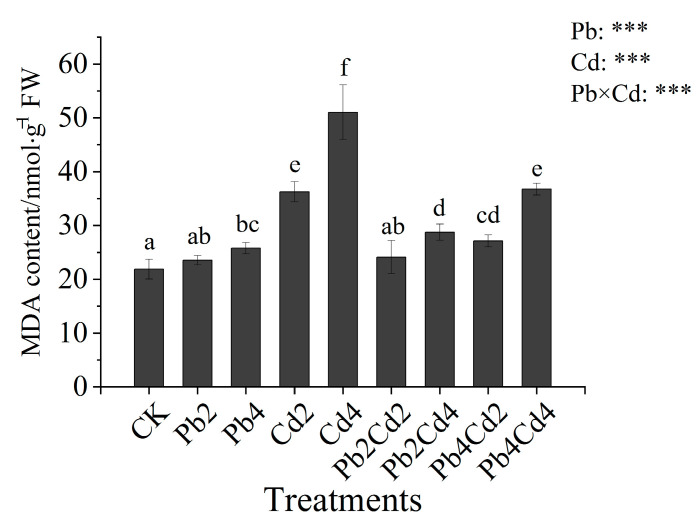
Effects of individual and combined Pb and Cd stress on malonaldehyde (MDA) content in *F*. *parvifolia*. The post hoc test used for the estimation of the significance level is the Duncan test. Different lowercase letters indicate significant differences at *p* < 0.05 between treatments. Each value represents mean values ± SEs (n = 5). ***, *p* < 0.001.

Both individual and combined Pb and Cd stress had significant impacts on leaf MDA content in *F*. *parvifolia* (*p* < 0.001) (Figure 1). Compared with CK, MDA content was increased by 17.72%, 65.62%, and 132.97% under the Pb4, Cd2, and Cd4 treatments, respectively. The observed increase under the Cd-treatment groups was larger than that under the Pb treatments. Moreover, MDA content under Pb and Cd combined stress was lower than that under individual Cd stress and higher than that under individual Pb stress. Among Pb and Cd combined treatments, the highest MDA content was observed in the Pb4Cd4 treatment, followed by the Pb2Cd4, Pb4Cd2, and Pb2Cd2 treatments.

**Figure 2:**
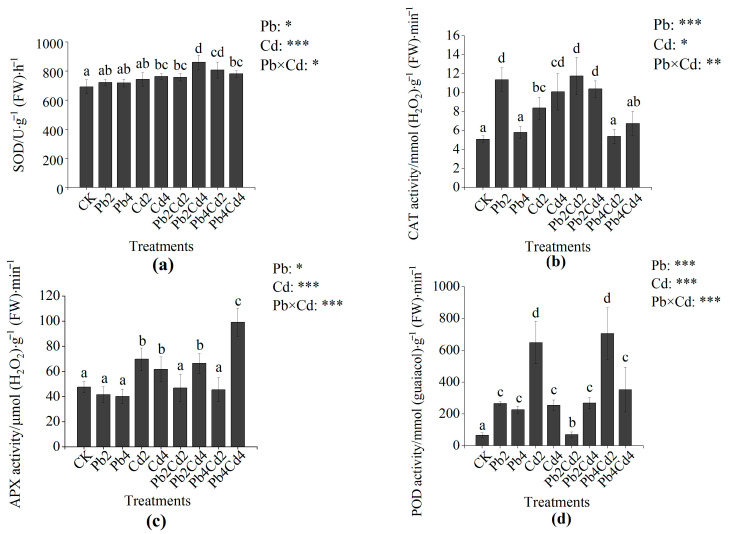
Effects of individual and combined Pb and Cd stress on the antioxidant enzyme activities in *F*. *parvifolia*. (**a**) The superoxide dismutase (SOD). (**b**) The catalase (CAT). (**c**) The ascorbate peroxidase (APX). (**d**) The peroxidase (POD). The post hoc test used for the estimation of the significance level is the Duncan test. Different lowercase letters indicate significant differences at *p* < 0.05 between treatments. Each value represents mean values ± SEs (n = 5). *, *p* < 0.05; **, *p* < 0.01; ***, *p* < 0.001.

Plants are capable of reducing MDA through an endogenous antioxidant system. The activity of POD under the Pb2 and Pb4 treatments was significantly increased by 301.78% and 242.85%, respectively, as compared with those under the CK treatment. CAT activity under the Pb2 treatment was significantly increased by 123.38% as compared with that under the CK treatment. In addition, SOD activity under high Cd stress was significantly higher than that under the CK treatment (Figure 2a), and the activities of CAT, APX, and POD under individual Cd treatments were all significantly increased as compared with those under the CK treatment (Figure 2b–d). Moreover, CAT and APX activities under the Pb4Cd2 treatment were significantly reduced by 35.80% and 34.61% as compared with those under the Cd2 treatment, respectively. Both SOD and APX activities under the Pb2Cd4 treatment were significantly increased by 19.09% and 59.66% as compared with those under the Pb2 treatment, respectively. APX activity was significantly increased by 59.66% and 146.67% under the Pb2Cd4 and Pb4Cd4 treatments compared to the Pb2 and Pb4 treatments, respectively. Moreover, the interaction between Pb and Cd had a significant effect on the overall activities of antioxidant enzymes (*p* < 0.001).

### 3.4. Pb and Cd Uptake, Accumulation, and Translocation Characteristics of F. parvifolia Seedlings under Pb and Cd Stress

**Figure 3:**
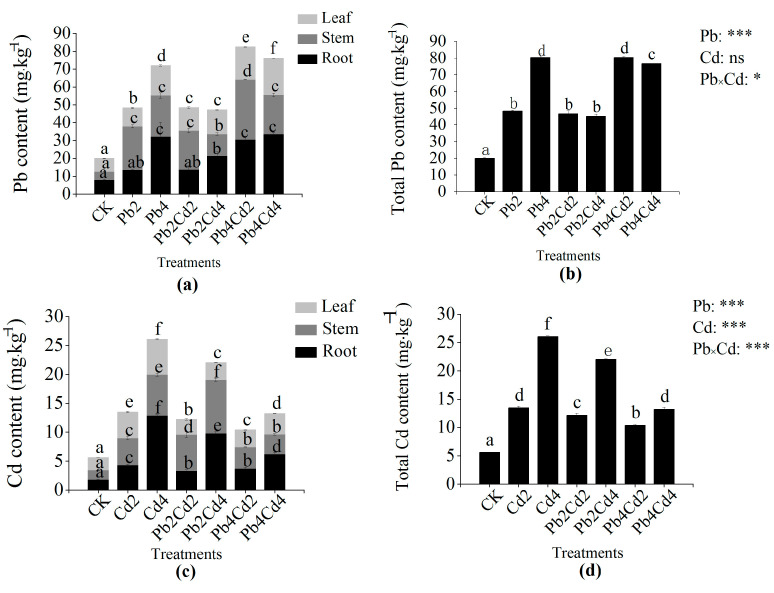
Effects of individual and combined Pb and Cd stress on Pb and Cd content of *F*. *parvifolia*. (**a**) The Pb content of plant different tissues. (**b**) The total Pb content in the plant. (**c**) The Cd content of plant different tissues. (**d**) The total Cd content in the plant. The post hoc test used for the estimation of the significance level is the Duncan test. Different lowercase letters indicate significant differences at *p* < 0.05 between treatments. Each value represents mean values ± SEs (n = 5). ns, *p* > 0.05; *, *p* < 0.05; ***, *p* < 0.001.

The Pb content of *F*. *parvifolia* in roots, leaves, and total biomass all significantly increased as Pb concentration increased (Figure 3a,b); similarly, the Cd content of plant in roots, stems, leaves, and total biomass exhibited an obvious upward trend as Cd concentration increased (Figure 3c,d). Moreover, Cd content was significantly reduced under combined treatments when compared to individual Cd treatments. The Cd content in plant roots and leaves under combined treatments significantly decreased as compared with those under individual Cd treatments. Conversely, the Pb content in plant roots under Pb and Cd combined treatments exhibited no obvious change when compared to that under the Pb treatments. However, the Pb content across the whole plant significantly declined under the Pb4Cd4 treatment when compared to that under the Pb4 treatment. 

**Figure 4:**
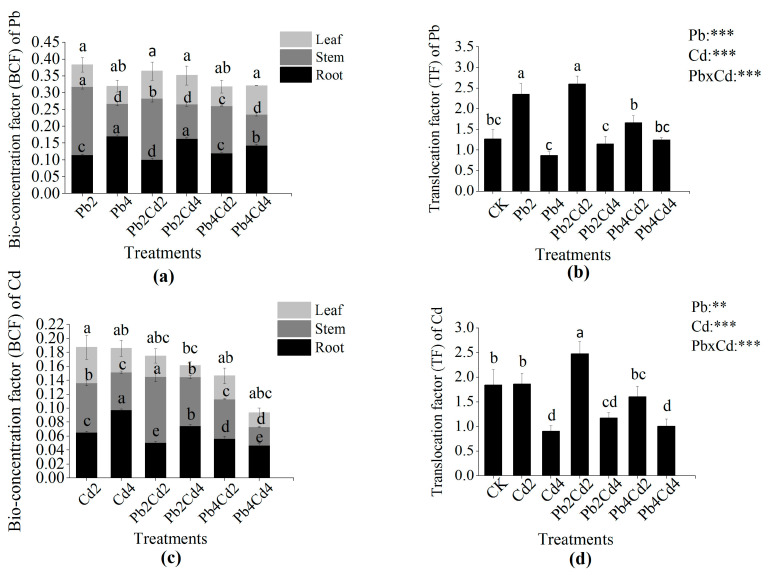
Effects of individual and combined Pb and Cd stress on Pb and Cd accumulation and translocation characteristics of *F*. *parvifolia*. (**a**) The bio-concentration (BCF) of Pb in plant different tissues. (**b**) The translocation (TF) of Pb in plant shoot. (**c**) The BCF of Cd in plant different tissues. (**d**) The TF of Cd in plant shoot. The post hoc test used for the estimation of the significance level is the Duncan test. Different lowercase letters indicate significant differences at *p* < 0.05 between treatments. Each value represents mean values ± SEs (n = 5). **, *p* < 0.01; ***, *p* < 0.001.

The accumulation and translocation characteristics are illustrated in Figure 4. It was found that the bio-concentration factor of Pb (BCF_Pb_) and Cd (BCF_Cd_) was lower than 1.0, while the translocation factor (TF) of Pb and Cd was higher than 1.0 except for Pb4 and Cd4 treatments. Interestingly, BCF_Pb_ and BCF_Cd_ across the whole plant decreased alongside a corresponding increase in the concentration of individual and combination treatments. Additionally, BCF_Pb_ in plant roots significantly increased with increasing Pb concentration, while BCF_Pb_ in plant stems significantly decreased alongside increased Pb concentration. In general, the variability of BCF_Cd_ across different plant tissues under individual Pb or Cd treatments was in agreement with that of BCF_Pb_. The TF of Pb and Cd under high concentrations of Pb and Cd significantly decreased when compared to that under low concentrations of Pb and Cd. BCF_Pb_ and BCF_Cd_ under combined Pb and Cd treatments were generally lower than that under individual treatments. Moreover, the BCF_Cd_ in roots was significantly reduced under combined Pb and Cd treatments in contrast to individual Cd treatments. The BCF_Pb_ and BCF_Cd_ in leaf tissue had no significant change under Pb and Cd combined stress when compared to individual Pb or Cd stress. The TF of Pb and Cd was significantly influenced by the interaction between Pb and Cd (*p* < 0.001).

### 3.5. Pearson Correlation Analysis of Cd Content, Accumulation, and Physiological Indexes

**Figure 5:**
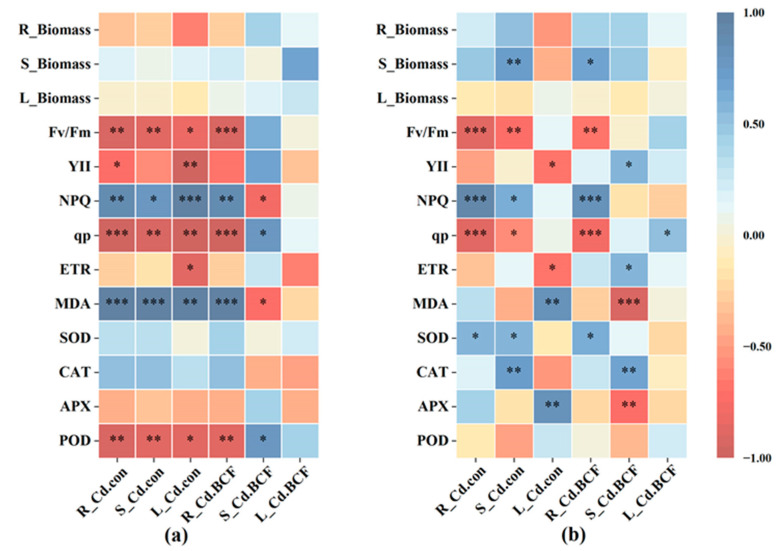
Heat map showing a correlation between Cd content, accumulation, and physiological index via Pearson correlation coefficients. (**a**) The correlation between Cd content, accumulation, and physiological index under individual Cd treatments. (**b**) The correlation between Cd content, accumulation, and physiological index under combined Pb and Cd treatments. R, root; S, stem; L, leaf; Fv/Fm, maximum photochemical efficiency; YII, actual photochemical efficiency; NPQ, non-photochemical quenching coefficient; qp, photochemical quenching coefficient; ETR, electron transport rate; MDA, malondialdehyde; SOD, superoxide dismutase; CAT, catalase; APX, ascorbate peroxidase; POD, guaiacol peroxidase; R_Cd.con, root Cd content; S_Cd.con, stem Cd content; L_Cd.con, leaf Cd content; R_Cd.BCF, root Cd bio-concentration factor; S_Cd.BCF, stem Cd bio-concentration factor; L_Cd. BCF, leaf Cd bio-concentration factor. *, *p* < 0.05; **, *p* < 0.01; ***, *p* < 0.001. The post hoc test used for the estimation of the significance level is the Duncan test.

The correlation between Cd content in plant tissues (root, stem, and leaf) and plant physiological indicators of *F*. *parvifolia* under different treatments is shown in Figure 5. Under individual Cd treatments, the Cd content in root, stem, and leaf tissues showed a significant (*p* < 0.05) positive correlation with NPQ and MDA but a negative correlation with POD and chlorophyll fluorescence parameters (except for NPQ, YII, and ETR). Moreover, the BCF_Cd_ of root tissue under individual Cd treatments showed a significant (*p* < 0.05) positive correlation with NPQ and MDA and a negative correlation with POD and chlorophyll fluorescence parameters (except for NPQ, YII, and ETR). However, the correlation between the BCF_Cd_ of stem tissue and physiological indexes was contrary to those observed in root tissue. Under the Pb and Cd combined treatments, the Cd content of root, stem, and leaf showed a significant (*p* < 0.05) positive correlation with biomass, MDA, and antioxidant enzyme activities (except for POD activity), while there was a negative correlation with chlorophyll fluorescence parameters. In addition, the bio-concentration factor of Cd in root was significantly (*p* < 0.05) associated with biomass, MDA, chlorophyll fluorescence parameters, and antioxidant enzyme activities (except for POD activity).

## 4. Discussion

### 4.1. The Effects of Pb and Cd on Biomass 

The visual influence of PTEs stress on plants is reflected in plant height and leaf. In this study, the biomass of different tissues (root, stem, and leaf) and the total had no significant change under individual Pb stress when compared to CK treatment, respectively. However, previous studies showed a reduction in fresh biomass of *Coriandrum sativum* under Pb stress [38]. The little change of biomass under individual Pb stress may be owing to the difference in plant species and Pb concentration. The biomass of different tissues and the total were significantly decreased under individual Cd stress as compared with CK treatment. Inhibition of biomass is owing to the disintegration of photosynthetic pigments and peroxidation of membrane lipids caused by Cd accumulation in plants [34]. Previous studies also demonstrated that the shoot dry biomass of potatoes was significantly decreased under Cd stress [3]. Interestingly, a few studies reported that a low concentration of Cd stimulates root growth [39]. The different results are associated with metal concentrations and plant species. Nevertheless, we found the biomass of different tissues under the Pb2Cd4 treatment was significantly higher than that under the Cd4 treatment, which demonstrated that the presence of a low concentration of Pb could alleviate phytotoxicity caused by a high concentration of Cd in plants and promote biomass production. Nonetheless, these results differed from our speculation that the adverse effect of combined treatments was greater than that of individual treatments with the same concentration. Redha et al. [9] also reported that the toxicity of metal mixtures (Pb, Cd, and Ni) was higher than that of individual metals. The reason for our study result may be related to root growth. Root and shoot growth are dependent upon one another, with roots providing water, minerals, and organic matter to the shoot and the shoot providing sugars, vitamins, and other nutrients to roots [40]. Roots directly contact and absorb matter in the soil, so they are relatively easily influenced by PTEs contamination in soil, leading to root structural and functional damage and nutrient deficiency in the aboveground parts. Meanwhile, our study results also demonstrated that Pb inhibited Cd accumulation in plant roots. Therefore, the presence of Pb decreases Cd phytotoxicity due to the reduction of Cd content in plant roots. Moreover, root, leaf, and total biomass under combined treatments were lower than that under individual Pb stress, which indicates that the presence of Cd aggravates Pb phytotoxicity in plants. This phenomenon could be attributed to Cd inhibiting the absorption of nutrient elements (Fe^2+^, Mg^2+^, Ca^2+^, and so on) [41].

### 4.2. The Effects of Pb and Cd on Photosynthesis

Gas exchange parameters can mirror the photosynthetic efficiency of plants. In this research, individual Pb stress improved the photosynthetic efficiency of plant leaves, and *G*_s_ and *T*_r_ also increased. This is an adaptive mechanism to Pb stress. Improving leaf photosynthetic efficiency because of the increase of carbon dioxide due to leaf stomatal opening [42]. Conversely, individual Cd stress inhibited leaf stomatal opening and transpiration rate, resulting in the reduction of photosynthetic efficiency. Moreover, *C*_i_ values decreased as a whole, thereby leading to a reduction in *P*_n_ value mainly due to the non-stomatal limiting factor [43], which was attributed to the cell membrane of stomata damage [44]. Additionally, the presence of Pb improved photosynthetic efficiency under individual Cd4 treatment, which may be because Pb enhances the enzyme activities involved in the chlorophyll synthesis pathway, thereby improving carbon dioxide assimilation efficiency [45]. However, the presence of Cd inhibited photosynthetic and transpiration rates as well as stomatal opening under individual Pb treatments. These may be due to stomatal closure caused by the interaction between Pb and Cd, thereby inhibiting normal transpiration and carbon assimilation.

The chlorophyll fluorescence parameters can reflect the damage degree of photosynthetic apparatus under PTEs stress [46]. In our experiment, we discovered that *F*_v_/*F*_m_, Y(II), *q*_p_, and ETR values under the Pb2 treatment were significantly decreased as compared with CK treatment. Moreover, our previous result demonstrated that chlorophyll a (Chl a) content significantly decreased and chloroplast structure was not damaged under the Pb2 treatment [47]. There is a strong correlation between chlorophyll fluorescence parameters and chlorophyll content. Furthermore, Pb inhibited the entry of nutrients to the root [42]. Therefore, this phenomenon is attributed to Chl, a degradation caused by nutrient deficiency. Nevertheless, individual Cd stress impeded the conversion of light energy to chemical energy and decreased the electron transport rate of photosystem II (PSII). This may be attributed to Cd affecting the reaction process in the Clavin cycle or photolysis system through manganese protein [48]. Moreover, NPQ significantly increased under individual Cd stress. These results indicated that PSII can convert the remaining excitation energy into heat energy and dissipate it through the lutein cycle [49]. In previous studies, the NPQ value also significantly increased in two varieties of maize seedlings under Cd stress [48]. Furthermore, the result of this study showed that the presence of Pb increased *F*_v_/*F*_m_, Y(II), *q*_p_, and ETR values under Cd stress. The result may be due to Pb inhibiting the entry of Cd to the root, reducing the chloroplast membrane damage, and promoting thylakoid accumulation and photochemical efficiency. Previous studies reported that spermidine (Spd) can promote the synthesis of ATP and the accumulation of thylakoids and increase the linear electron flow rate (LEF) and the photochemical efficiency of PSII [50].

### 4.3. The Effects of Pb and Cd on MDA Content and Antioxidant Enzymes

PTEs can accelerate the production of reactive oxygen species (ROS), and the accumulation of ROS to a certain amount can result in oxidative stress, which leads to membrane lipid peroxidation and the production of MDA [51]. Results showed that MDA content significantly increased under Pb4, Cd2, and Cd4 treatments when compared to CK treatment, and the increase of MDA content under individual Cd treatments was higher than that under individual Pb treatments. These results indicated that Pb and Cd stress could trigger oxidative stress. Furthermore, Cd phytotoxicity in *F. parvifolia* seedlings was higher than Pb. The reason might be that the use of chloride as the Cd stress source aggravated Cd toxicity [52]. Earlier reports also found that MDA and H_2_O_2_ content increased significantly under Pb and Cd stress [38,53]. Moreover, the presence of Pb significantly inhibited the accumulation of MDA under individual Cd treatments. This phenomenon was attributed to the antagonism effect under Pb and Cd combined treatments. Furthermore, Pb inhibited the entry of Cd to the plant and reduced the damage degree of the cell membrane. Nonetheless, plants could reduce MDA content via an antioxidant system [54]. ROS mainly includes superoxide anion (O_2_^−^), singlet oxygen (^1^O_2_), hydrogen peroxide (H_2_O_2_), and the hydroxyl radical (OH) [55]. Antioxidant enzymes include SOD, CAT, APX, and POD. SOD can catalyze the conversion of O_2_^−^ into O_2_ and H_2_O_2_, and CAT and POD can further catalyze H_2_O_2_ into H_2_O [54,56]. In the present study, compared with CK, POD and CAT activities were significantly increased under the Pb2 treatment. However, POD activity was significantly increased, and CAT activity had no different change under the Pb4 treatment. This result suggested that POD played a predominant role in reducing the accumulation of ROS and also inhibited CAT activity to a certain extent. It is consistent with the change of POD and CAT activities under Cd stress [54]. However, CAT, APX, and POD activities significantly increased under individual Cd treatments, and SOD activity significantly increased under the Cd4 treatment as compared with CK treatment, respectively. It indicates that antioxidant enzymes cooperate to remove ROS. Kumar et al. [57] also reported that SOD, APX, glutathione reductase (GR), glutathione (GSH), and ascorbic acid (AsA) activities increased under chromium (Cr (VI)) stress. In addition, the presence of Pb increased SOD activity under individual Cd treatments, which can attribute to Pb upregulating the expression of genes relevant to SOD [58]. Similar to this result, Lanza et al. [59] reported that selenium (Se) alleviated oxidative stress caused by Cd via increasing antioxidant enzyme activities. 

### 4.4. The Effects of Pb and Cd on Pb and Cd Accumulation and Translocation Characteristics of F. parvifolia

Non-essential elements (Pb, Cd, and Hg) in the soil can be absorbed by plants via transporters and ion channels [3] and subsequently accumulate or be transported to various parts of the plant, thereby providing the possibility of remediating contaminated soil via the plant. Our experiment demonstrated that the presence of Pb could reduce Cd content in plant roots and leaves under individual Cd treatments. At the same time, BCF of Cd in the root also significantly decreased under individual Cd treatments when compared to Pb and Cd combined treatments. Shahid et al. [3] also found the application of Se reduced the Cd and arsenic (As) content of potatoes in the root, leave, and stolon. These results may be due to the antagonism effect between Pb and Cd on root binding sites and inhibition of the absorption of Cd by the plant [60]. A previous study reported that the Cd uptake by the plant was closely related to iron-regulated transporter 1 (OsIRT1) and natural resistance-associated macrophage protein 1 (OsNramp1) [61,62]. Cd is absorbed by the plant through Ca^2+^, Mg^2+^, and Fe^2+^ channels and competes with other divalent cations [63]. Therefore, the presence of Pb might have disrupted iron and other divalent cation channels and transporters, leading to inhibition in Cd entry to the plant root. According to the result of correlation analysis, we found another reason related to the change of SOD and POD activities and stem biomass (Figure 5). We observed that the presence of Cd could reduce the content and BCF of Pb in plants under Pb and Cd combined treatments when compared to individual Pb treatments, which was not the same as our hypothesis. This phenomenon is similar to the result that a high concentration of Cd reduced Pb accumulation in the stem and leaf of potatoes and wheat [64]. This might be attributable to the competition of Pb and Cd ions [65]. On the other hand, metal accumulation and transfer were also associated with transporter proteins and ATPase [66]. Chen et al. [67] revealed that Cd accumulation was reduced in rice due to decreased expression of Cd transporters; previous research has found that metal-transporting ATPase could absorb and transport non-essential metal ions via ATP hydrolysis [66]. In the case of metal distribution, many scholars have demonstrated that Pb and Cd were mostly retained in plant roots [68,69]. However, we observed that Pb and Cd were concentrated in plant shoots under all treatments. The reason for this may be due to the different responses of the plant to Pb and Cd stress. A large number of previous studies showed that the BCF and TF of Cd were higher than those of Pb [45,60]. However, in our study, the BCF and TF of Cd were lower than those of Pb. This may be because Cd toxicity was significantly greater than Pb toxicity, resulting in plant growth and physiology damage and metabolic disorders under Cd stress, thereby influencing the normal capacity of metals enrichment by the plant [60]. Another reason was related to the mobility and bioavailability of Pb and Cd ions. The use of soluble nitrate salt as a Pb source could also increase Pb mobility [52]. The mobility and bioavailability of Pb and Cd are also influenced by pH value, organic matter, and soil properties [70]. The available Cd concentration was significantly positively related to soil pH, cation exchange capacity, and the proportion of illite and illite–smectite mixed layers of clay minerals, and the available Pb concentration was significantly positively related to soil pH and organic matter [71]. Liu et al. [72] found that the adsorption of selenite on pyrite particles could reduce Se content to a certain extent. The pyrite particles could remediate technetium (Tc)-contaminated soil [73]. Wang et al. [74] also found that synthetic pyrite particles might reduce the bioavailable amount of Cr (VI) in contaminated water or soil. Furthermore, the BCF values of Pb and Cd were lower than 0.5, while the TF values of Pb and Cd were greater than 1.0 except for Pb4 and Cd4 treatments. It indicates that Pb and Cd mainly transfer to the shoot, but the Pb and Cd accumulation capacity of *F. parvifolia* is low. The result can be attributed to the detoxification mechanism of *F. parvifolia*’s exclusion of Pb and Cd as well as the transporter protein of Pb and Cd. Furthermore, our results showed that the Pb content was higher than Cd content in all treatments, which is consistent with the Cr (VI) content being lower than Pb content in an area affected by mining in China [75]. Additionally, BCF and TF values can indicate whether the plant is a hyperaccumulator or not. Criteria for being a hyperaccumulator plant include BCF and TF values greater than 1.0, respectively [76]. Consequently, the result of the present study suggests that *F. parvifolia* is not a hyperaccumulator plant, but it has a high tolerance to Pb.

## 5. Conclusions

In this study, we could conclude that *F*. *parvifolia* seedlings have a strong tolerance to individual Pb stress, while they were damaged by individual Cd stress. Furthermore, there was an antagonism effect between Pb and Cd. The presence of Pb could alleviate Cd phytotoxicity in the plant physiology process by inhibiting Cd uptake and accumulation, improving photosynthesis efficiency and antioxidant enzyme activities. The order of phytotoxicity in different treatments is Cd> Pb + Cd> Pb. Based on our study result, we should further investigate how the interaction between Pb and Cd affects their mobility and bioavailability to efficiently remediate Pb and Cd combination pollution. Moreover, phytoremediation technology may cause secondary contamination, so the future study orientation should be focused on how to reduce toxic matter output during utilizing phytoremediation technology.

## Figures and Tables

**Table 1:** Physicochemical characteristics of the experimental soil.

Soil Characteristic	Value
pH	6.90 ± 0.01
Organic matter	156.50 ± 0.56 g·kg^−1^
Organic carbon	90.80 ± 0.32 g·kg^−1^
Total nitrogen	1.25 ± 0.02 g·kg^−1^
available phosphorus	10.59 ± 0.10 g·kg^−1^
Total potassium	8.24 ± 0.17 g·kg^−1^
Pb content	23.17 ± 1.04 mg·kg^−1^
Cd content	0.45 ± 0.02 mg·kg^−1^

**Table 2:** Experimental design.

Treatments	Pb Concentration (mmol·L^−1^)	Cd Concentration (mmol·L^−1^)
CK	0	0
Pb2	2	0
Pb4	4	0
Cd2	0	2
Cd4	0	4
Pb2Cd2	2	2
Pb2Cd4	2	4
Pb4Cd2	4	2
Pb4Cd4	4	4

**Table 3:** Effects of individual and combined Pb and Cd stress on the biomass of different *F. parvifolia* tissues. The post hoc test used for the estimation of the significance level is the Duncan test. Different lowercase letters in the same column indicate significant differences at *p* < 0.05. Each value represents mean values ± SEs (n = 5). ns, *p* > 0.05; *, *p* < 0.05; **, *p* < 0.01; ***, *p* < 0.001.

Treatments	Root Biomass (g)	Stem Biomass (g)	Leaf Biomass (g)	Total Biomass (g)
CK	5.08 ± 0.04bc	2.96 ± 0.49c	1.10 ± 0.45de	9.14 ± 0.82d
Pb2	6.08 ± 0.33c	2.73 ± 0.66bc	0.95 ± 0.20cd	9.77 ± 0.93d
Pb4	5.00 ± 1.02bc	2.74 ± 0.65bc	1.33 ± 0.35e	9.07 ± 1.65d
Cd2	3.08 ± 0.37a	1.89 ± 0.55ab	0.28 ± 0.12a	5.25 ± 0.85ab
Cd4	2.81 ± 0.56a	2.02 ± 0.57ab	0.27 ± 0.09a	5.07 ± 0.93a
Pb2Cd2	5.11 ± 0.59bc	2.27 ± 0.48abc	0.54 ± 0.16ab	7.92 ± 1.18bc
Pb2Cd4	5.23 ± 1.42bc	3.03 ± 0.57c	0.71 ± 0.18bc	8.97 ± 1.70d
Pb4Cd2	4.30 ± 1.60ab	2.38 ± 0.34abc	0.81 ± 0.20bc	7.48 ± 2.03bcd
Pb4Cd4	3.64 ± 1.01ab	1.79 ± 0.33a	0.64 ± 0.31ab	6.07 ± 1.13abc
Pb	**	ns	*	**
Cd	*	*	***	***
Pb × Cd	ns	ns	ns	ns

**Table 4:** Effects of individual and combined Pb and Cd stress on photosynthetic characteristics in *F*. *parvifolia.* The post hoc test used for the estimation of the significance level is the Duncan test. Different lowercase letters in the same column indicate significant differences at *p* < 0.05. Each value represents mean values ± SEs (n = 5). ns, *p* > 0.05; *, *p* < 0.05; **, *p* < 0.01; ***, *p* < 0.001.

Treatments	*P*_n_ (μmol·m^−2^·s^−1^)	*G*_s_ (mol·m^−2^·s^−1^)	*C*_i_ (μmol·mol^−1^)	*T*_r_ (mol·m^−2^·s^−1^)
CK	6.27 ± 1.02c	0.126 ± 0.013de	296.88 ± 6.27bc	2.72 ± 0.21d
Pb2	7.89 ± 0.11d	0.137 ± 0.002e	281.38 ± 0.69b	2.70 ± 0.05d
Pb4	8.40 ± 0.92d	0.197 ± 0.020f	305.48 ± 2.62bc	3.55 ± 0.28e
Cd2	4.78 ± 1.43b	0.109 ± 0.023cd	311.37 ± 11.81c	1.80 ± 0.42bc
Cd4	2.99 ± 1.07a	0.052 ± 0.022a	282.84 ± 16.92b	0.90 ± 0.33a
Pb2Cd2	4.21 ± 1.01ab	0.088 ± 0.010bc	301.86 ± 20.49bc	2.03 ± 0.19c
Pb2Cd4	4.09 ± 0.21ab	0.072 ± 0.007ab	289.81 ± 5.54bc	1.65 ± 0.14bc
Pb4Cd2	5.28 ± 1.40bc	0.068 ± 0.033ab	244.30 ± 24.89a	1.59 ± 0.60bc
Pb4Cd4	4.66 ± 0.83b	0.060 ± 0.012ab	251.45 ± 29.92a	1.48 ± 0.25b
Pb	**	ns	***	*
Cd	***	***	*	***
Pb × Cd	ns	***	***	***

**Table 5:** Effects of individual and combined Pb and Cd stress on chlorophyll fluorescence parameters in *F*. *parvifolia.* The post hoc test used for the estimation of the significance level is the Duncan test. Different lowercase letters in the same column indicate significant differences at *p* < 0.05. Each value represents mean values ± SEs (n = 5). ns, *p* > 0.05; *, *p* < 0.05; **, *p* < 0.01; ***, *p* < 0.001.

Treatments	*F*_v_/*F*_m_	Y(II)	*q*_p_	NPQ	ETR
CK	0.7290 ± 0.0164d	0.6363 ± 0.0168d	0.9218 ± 0.0062e	0.3163 ± 0.0177a	16.85 ± 0.40f
Pb2	0.7034 ± 0.0144bc	0.5954 ± 0.0184c	0.8983 ± 0.0075d	0.2989 ± 0.0269a	15.25 ± 0.46e
Pb4	0.7220 ± 0.0338cd	0.6090 ± 0.0239cd	0.8990 ± 0.0298d	0.3085 ± 0.0550a	16.26 ± 1.09f
Cd2	0.6883 ± 0.0209ab	0.5558 ± 0.0124b	0.8584 ± 0.0281b	0.2997 ± 0.0328a	13.15 ± 0.54b
Cd4	0.6685 ± 0.0303a	0.5036 ± 0.0532a	0.8262 ± 0.0443a	0.5036 ± 0.1163b	12.18 ± 1.12a
Pb2Cd2	0.7253 ± 0.0241cd	0.5891 ± 0.0097c	0.8633 ± 0.0471bc	0.2772 ± 0.0279a	14.08 ± 0.50cd
Pb2Cd4	0.6998 ± 0.0333bc	0.5861 ± 0.0345c	0.8553 ± 0.0313b	0.3074 ± 0.0495a	13.96 ± 0.19cd
Pb4Cd2	0.7323 ± 0.0176d	0.6019 ± 0.0404c	0.8915 ± 0.0198cd	0.2728 ± 0.0310a	14.48 ± 0.71d
Pb4Cd4	0.7003 ± 0.0269bc	0.5173 ± 0.0602a	0.8636 ± 0.0366bc	0.2889 ± 0.0154a	13.48 ± 0.85bc
Pb	*	*	ns	ns	*
Cd	***	***	*	***	***
Pb × Cd	**	***	ns	*	***

## Data Availability

The datasets used or analyzed during the current study are available from the corresponding author upon reasonable request.
